# Evidence of anticipatory immune and hormonal responses to predation risk in an echinoderm

**DOI:** 10.1038/s41598-021-89805-0

**Published:** 2021-05-21

**Authors:** Jean-François Hamel, Sara Jobson, Guillaume Caulier, Annie Mercier

**Affiliations:** 1 Society for the Exploration and Valuing of the Environment (SEVE), St. Philips, NL A1M 2B7 Canada; 2 Department of Ocean Sciences, Memorial University, St. John’s, NL A1C 5S7 Canada

**Keywords:** Behavioural ecology, Innate immunity, Neuroimmunology, Animal behaviour, Evolutionary ecology

## Abstract

Recent efforts have been devoted to the link between responses to non-physical stressors and immune states in animals, mostly using human and other vertebrate models. Despite evolutionary relevance, comparatively limited work on the appraisal of predation risk and aspects of cognitive ecology and ecoimmunology has been carried out in non-chordate animals. The present study explored the capacity of holothuroid echinoderms to display an immune response to both reactive and anticipatory predatory stressors. Experimental trials and a mix of behavioural, cellular and hormonal markers were used, with a focus on coelomocytes (analogues of mammalian leukocytes), which are the main components of the echinoderm innate immunity. Findings suggest that holothuroids can not only appraise threatening cues (i.e. scent of a predator or alarm signals from injured conspecifics) but prepare themselves immunologically, presumably to cope more efficiently with potential future injuries. The responses share features with recently defined central emotional states and wane after prolonged stress in a manner akin to habituation, which are traits that have rarely been shown in non-vertebrates, and never in echinoderms. Because echinoderms sit alongside chordates in the deuterostome clade, such findings offer unique insights into the adaptive value and evolution of stress responses in animals.

## Introduction

Stress has been defined in various ways but can be viewed as the response of an organism subjected to a challenge that may result in real or possible danger to its integrity^1^. Stressors may be distinguished based on whether they are reactive (direct challenge to homeostasis, like an injury or predator attack) or anticipatory (perceived threat requiring cognitive appraisal, like a cue of predation risk)^2^. While fear in animals is in response to the former, anxiety is in response to the latter^3^. The stress response to both situations may involve a wide range of mechanisms, including changes in genetic, metabolic, energetic, immune, endocrine, neural and behavioural processes aiming to overcome and compensate for the imbalances produced by the stressor. With these reactions, the animal tries to avoid dangerous situations and any threats to its survival or integrity and ultimately to reintegrate a state of balance^1^.

While perceptible physical stressors (direct challenges) elicit a direct stress response, non-physical stressors first need to be appraised before eliciting a response^4^. Current knowledge on appraisal of non-physical stressors is almost entirely based on work conducted on humans (e.g. emotional/psychological stress) and in other members of the Chordata phylum (mammals and fishes), including in the context of predation risk^5^. More basal animals (non-vertebrates) are often not considered to have the necessary neural requirements to trigger anticipatory reactions; instead they are assumed to undergo strictly sensorimotor responses^6^. However, there is increasing evidence to support a re-evaluation of this assumption^7,8^, circling back to Darwin’s initial suggestion that insects have emotions^9^. For instance, research has shown that members of the phyla Arthropoda (insects, malacostracan decapods) and Mollusca (gastropods) may exhibit a variety of cognitive phenomena that were previously thought to be restricted to vertebrates or even to be unique to humans^8^. A new framework has been proposed for studying emotions across all animal species, based on a central emotion state with properties that are expressed through cognitive, behavioural, physiological and subjective components^10^. This approach disentangles emotions and their precursors from feelings (the conscious experience of emotional reactions), and moves away from comparisons with humans in favour of defining common features like scalability, persistence, valence and generalization to multiple contexts. While measuring cognitive responses in non-vertebrates represents a challenge^11^, the last decade has seen breakthroughs. For instance, aversive taste stimuli were suggested to elicit behavioural reactions analogous to conditioned fear in pond snails^12^ and cognitive bias was shown in honey bees exposed to vigorous shaking (mimicking danger), which subsequently interpreted ambiguous olfactory cues in a ‘pessimistic’ way similar to negative emotional states seen in vertebrates^13^. Similarly, bumblebees were shown to exhibit cognitive bias analogue to optimism^14^.

Interpreting such reactions in slow-moving aquatic organisms with few recognizable features (no eyes or limbs) relies on the development of suitable behavioural, physiological, or immunological proxies, which may be explored in the context of adaptive stress responses. The fight or flight response first described 80 years ago^15^, also called acute stress response, is triggered by a perceived (not necessarily actual) harmful event, attack, or threat to survival, to prepare the animal for fighting or fleeing^16^. A chain of reactions inside the body mobilises resources to deal with threatening circumstances, i.e. releasing hormones like adrenalin and cortisol, speeding the heart rate, slowing digestion, shunting blood flow to major muscle groups, giving the body a burst of energy and strength^15^. Burnovicz, et al.^17^ studied the cardiac response of a crab exposed to various stimuli: light pulse, air puff, virtual looming and a real visual danger. The first two did not trigger observable behaviour, but the last two elicited a clear escape response and a change in heart rate upon sensory stimulation. The correlation found between escape and cardiac responses supports that the crab triggered several defensive reactions in the face of impending danger^17^.

Where physiological metrics like cardiac responses cannot be measured (in the absence of a heart or circulatory system), immune responses provide a promising alternative^18^. Interestingly, Höglund, et al.^19^ showed that regulatory T cells (lymphocytes that control the activity of other types of immune cell), appear to increase sharply in number in response to psychological stress in humans. Terrestrial and aquatic invertebrates exhibit innate immunity comprised of cell-mediated phagocytosis, activation of cellular responses, and production of humoral antimicrobial compounds^20^. Their immune system is based on the presence of a group of cells called coelomocytes, which are abundantly described in the literature from different taxa (see reviews^21–24^) as the first line of defence, with their number and type varying dramatically during infections or following injury. Coelomocytes are found in the cavities of all echinoderm classes, including in the perivisceral coelom, the hydrovascular system, and the haemal system, as well as in the connective tissues and amongst various organs^25,26^. Their functions are similar to their leukocyte analogues in the immune system of vertebrates, such as formation of cellular clots, phagocytosis, encapsulation and clearance of parasites, bacteria and other foreign materials^27,28^. Encapsulation in echinoderms has been documented in classes Ophiuroidea, Echinoidea, and Holothuroidea, and its products are conventionally referred to as brown bodies or aggregates^29–31^. The latter are composed of various coelomocytes that can have different functions^22^. Based on work conducted on two echinoids (sea urchins), coelomocytes are considered sensitive biomarkers of marine environmental stress^32^. Apart from the immune response, the hormonal response offers other potential biomarkers of the stress response. For instance, cortisol is a steroid often referred to as the ‘stress hormone’ that is well known in aquatic vertebrates such as fishes^33^. It is also detected in non-chordate groups, including echinoderms^34^, and a spike in cortisol was recently determined to be an expression of stress in bivalves^35^ and holothuroids^36,37^.

The present study used levels of coelomocytes and cortisol to explore the capacity of holothuroid echinoderms, more precisely the species *Cucumaria frondosa*, to display an immune response to both reactive and anticipatory stressors (physical attack vs cues of predation risk). The approach mixed behavioural, immune and hormonal markers to test the following hypotheses: (1) if acute predation-related stress enhances the immune response, coelomocyte counts and cortisol levels will increase upon short-term acute exposure (˂3 h) to a threat; (2) if appraisal and perception of imminent threat is enough to trigger the stress response, direct contact with the stressor (predator) will not be necessary; (3) when the non-physical cue is prolonged without the threat (attack) materializing, chronic stress will translate into a decrease of coelomocyte counts back to baseline levels (i.e. habituation). Ultimately, the study seeks to build the knowledge base on cognitive ecology by assessing whether non-vertebrate deuterostomes can react to potential threats before they materialize by preparing their immune line of defence in anticipation. Identifying markers of non-physical stress in a basal deuterostome clade that shares closes ancestors with chordates^38^ provides an exciting tool for studying the adaptive value and early evolution of stress responses like anxiety inside the animal kingdom.

## Results

### Chronic exposure to direct and indirect stressors

**Figure 1:**
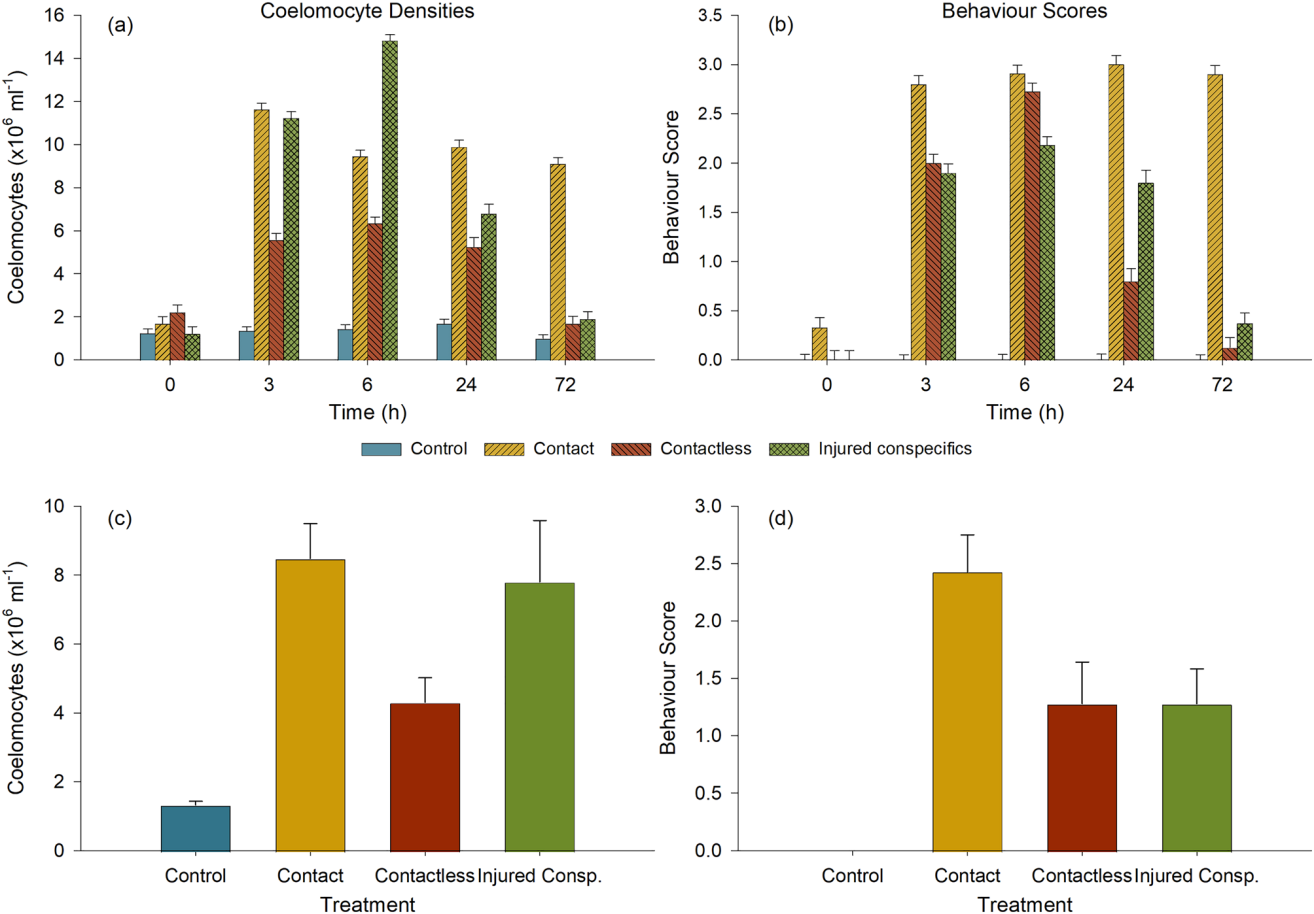
Immune and behavioural responses of *Cucumaria frondosa* to various treatments, including no stimulus (control), direct contact with the predator (contact), chemical signature of the predator (contactless), and chemical signature of injured conspecific (injured consp.). (**a**) Density of coelomocytes in the Polian vesicle and (**b**) corresponding behavioural score over 72-h exposure. Panels (**c**) and (**d**) illustrate the same metrics integrated over the duration of the experiment (72 h) for each treatment. Values in (**a**) and (**b**) are presented as mean ± S.E. (*n* = 3–9) and values in (**c**) and (**d**) are presented as mean ± 95% CI (*n* = 15–45).

All controls across treatments showed a baseline density of coelomocytes (all types combined) between 1.0 and 1.7 × 10^6^ cells ml^−1^ from the beginning to the end of the 72-h trial (global mean of 1.4 × 10^6^ cells ml^−1^; Fig. 1a). The control holothuroids remained firmly attached to the substrate (no displacement) with their tentacles extending periodically in the water column for feeding, corresponding to a resting state and a behaviour score < 0.3 (Fig. 1b).

In experiment 1, representing direct physical contact with the predatory asteroid (including predatory attacks), the total density of free coelomocytes rose from baseline values at time 0 to 11.6 × 10^6^ cells ml^−1^ after 3 h, representing an increase of about 730% (Fig. 1a). Coelomocyte densities decreased after 6 h but remained higher than baseline, between 9–10 × 10^6^ cells ml^−1^, until the end of the trial (Fig. 1a). From the onset (≤ 10 min), the exposed holothuroids displayed an increase in locomotory movement (92 cm min^−1^) and the typical escape response (detachment, rolling and muscular contractions), occasionally combined with active buoyancy adjustment (ABA)^39^, until the end of the experiment (72 h), corresponding to a mean behaviour score of 2.8–3.0 (Fig. 1b).

In experiment 2 (contactless treatment), holothuroids received only the chemical cue (i.e. ‘scent’) from the predator without any physical or visual cue (no actual physical threats for their physical integrity). The density of coelomocytes rose to 5.6 × 10^6^ cells ml^−1^ after 3 h, representing about 300% increase from baseline, which was lower than upon direct contact with the predator (Fig. 1a). Coelomocyte densities remained comparably high at 6.3 × 10^6^ cells ml^−1^ and 5.3 × 10^6^ cells ml^−1^ after 6 and 24 h, respectively, before falling back to baseline values below 1.7 × 10^6^ cells ml^−1^ after 72 h (Fig. 1a). The behaviour of holothuroids exposed to the predator scent was similar to the one recorded following direct contact during the first 6 h, i.e. rapid displacement (80–85 cm min^−1^), escape response and retraction of the tentacles, yielding a mean behaviour score of 2.7 (Fig. 1b). However, an increasing number of individuals returned to a resting state after 24 h, whereby a more static posture was adopted (decreased locomotion to ≤ 10 cm min^−1^, tentacles periodically extended, and no escape response), with a mean behaviour score of 0.8 (Fig. 1b). After 72 h, all exposed holothuroids had resumed normal behaviour (mean score of 0.1; Fig. 1b).

Experiment 3 (exposure to the scent of an injured conspecific) produced intermediate results, with overall sharper responses, but an eventual return to baseline. After 3 h of exposure, the density of coelomocytes rose sharply to 11.2 × 10^6^ cells ml^−1^, comparable to values in the treatment involving contact with the predator (about 700% increase; Fig. 1a). However, values continued to rise, reaching 14.8 × 10^6^ cells ml^−1^ after 6 h, which was higher than in the response to direct contact with the predator. Coelomocyte densities decreased to 6.8 × 10^6^ cells ml^−1^ after 24 h, mirroring values of the contactless treatment, before returning to values close to baseline, i.e. 1.9 × 10^6^ cells ml^−1^, after 72 h (Fig. 1a). Similar to the contactless treatment, behaviour scores rose markedly to 1.8–2.2 after 3–24 h and decreased back to near-baseline values (score of 0.4) after 72 h (Fig. 1b) and displacement peaked between 71 and 88 cm min^−1^.

Statistical analysis highlighted the interaction between the effects of treatment and time on coelomocyte density (*F*_12,252_ = 112.4, *p* < 0.001) and behaviour score (*F*_12,252_ = 84.5, *p* < 0.001). Further analyses conducted independently for each factor confirmed the clear effect of time (*F*_4,38_ = 23.2, *p* < 0.001); with spikes in coelomocyte density between 3 and 24 h followed by a return to baseline after 72 h in all treatments, except contact with the predator (Fig. 1a). An identical pattern was also statistically clear in the behaviour scores (Fig. 1b; *H* = 42.4, *df* = 4, *p* < 0.001). A clear effect of treatment on the coelomocyte density (Fig. 1c; *H* = 137.3, *df* = 3, *p* < 0.001) and behaviour score (Fig. 1d; *H* = 162.6, *df* = 3, *p* < 0.001) was also shown. When integrated over the 72-h response, coelomocyte densities deviated from controls inside treatments, but were similar among treatments; whereas behaviour scores showed full pairwise differences, except between the two contactless treatments (scent of predator and scent of injured conspecifics).

### Short-term contactless exposure to predator

**Figure 2:**
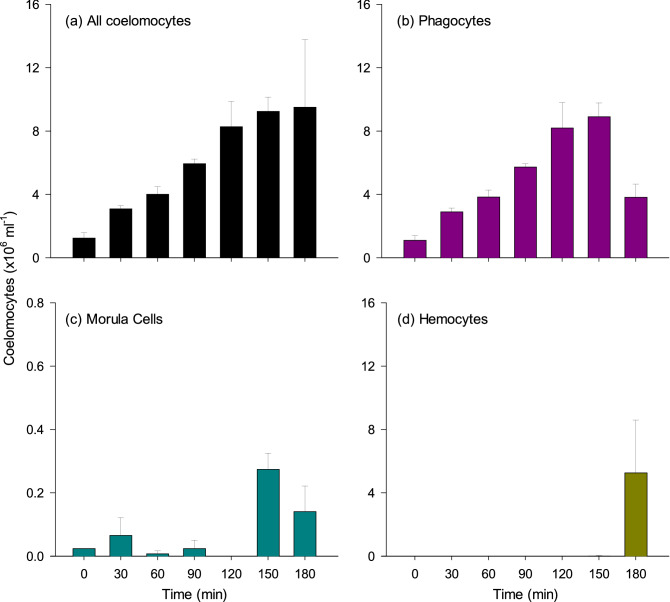
Immune response of *Cucumaria frondosa* to contactless exposure to the chemical signature of a predator over 180 min (3 h). Coelomocyte density was measured in the hydrovascular fluid of the Polian vesicle. (**a**) Global density of free coelomocytes (all types pooled). (**b**) Density of phagocytes. (**c**) Density of morula cells. (**d**) Density of hemocytes. Data presented as mean ± S.E. (*n* = 3). Note the different y-axis scale in (**c**).

From 0 to 150 min, the total number of free coelomocytes increased steadily by steps, from 1.3 × 10^6^ cells ml^−1^ to a maximum of 9.2 × 10^6^ cells ml^−1^ after 150 min (an increase of 770%), which was maintained after 180 min (Fig. 2a). Upon examination of coelomocyte types, a progressive increase in phagocytes was detected during the first 150 min, from 1.3 to 8.9 × 10^6^ cells ml^−1^, followed by a decrease over the next 30 min to 3.8 × 10^6^ cells ml^−1^ after 180 min (Fig. 2b). The morula cells remained low until 120 min (≤ 0.2 × 10^6^ cells ml^−1^) and increased to > 0.2 × 10^6^ cells ml^−1^ after 150 min, before decreasing back to 0.1 × 10^6^ cells ml^−1^ after 180 min (Fig. 2c). Moreover, the hemocyte counts increased drastically from near zero to 5.3 × 10^6^ cells ml^−1^ after 180 min (an increase of ~ 2500%; Fig. 2d). After 180 min, the hemocytes represented 20–93% of all coelomocytes present (Fig. 2d), at a time when phagocyte counts were decreasing back to baseline values in some individuals. The statistical analysis of coelomocyte density (pooled types) confirmed the clear effect of time (*F*_6,14_ = 3.36, *p* = 0.029), including a clear departure from baseline (time 0) after 150 min and 180 min (post-hoc test, *p* = 0.032 and 0.031, respectively). Phagocytes also displayed a clear stepwise increase (*F*_6,14_ = 13.07, *p* < 0.001), with departure from baseline becoming clear from 90 min onward (post-hoc test, *p* < 0.004). Morula cells peaked at 150 min (Fig. 2c; post-hoc test, *p* = 0.006) and hemocytes peaked at 180 min (Fig. 2d; post-hoc test, *p* = 0.004).

Individuals in the control group and those assessed at time 0 exhibited cloacal opening rates (respiration) around 1 min^−1^ while their force of attachment to the substrate was ~ 14.7 N. All remained firmly attached to the substrate and ~ 65% of them had their tentacles extended at any time point. After 30 min of exposure to the stressor, cloacal openings increased to 4.3 min^−1^, reaching a maximum of 7 min^−1^ after 60 min, thereafter remaining steady until the end (180 min). The force of attachment to the substrate dropped to ~ 0.2 N after 30 min and to almost 0 N thereafter. Concurrently, the locomotor activity increased from 0 to 35 cm min^−1^ inside the first 60 min, and up to 80 cm min^−1^ after 90 min, with 46% of individuals showing the typical escape response. Some 33% of individuals showed excessive production of mucus on the surface of the body wall after 60 min and some of them were also showing clear ABA after 120 min.

**Figure 3:**
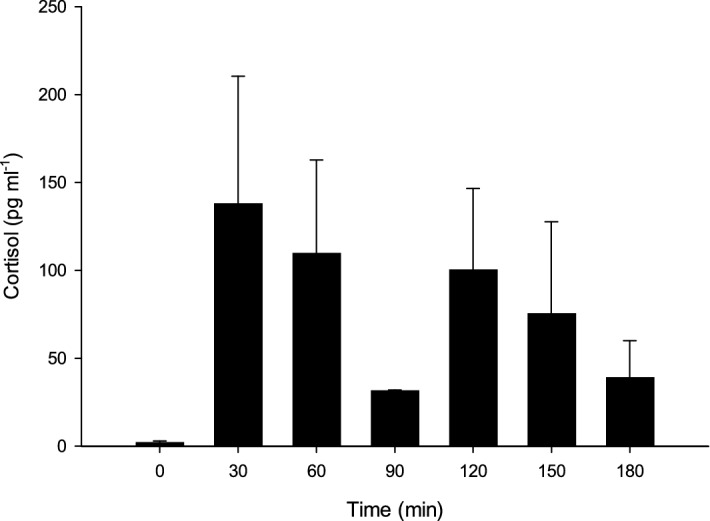
Hormonal response of *Cucumaria frondosa* to contactless exposure to the chemical signature of a predator over 180 min (3 h). Cortisol concentration in pg ml^−1^ (mean ± SE; *n* = 2–6) was measured in the hydrovascular fluid of the Polian vesicle.

Individuals in the short-term exposure to the scent of the predator showed levels of cortisol that spiked after 30 min at levels that were clearly higher than at time 0 (*H* = 13.31, *df* = 6, *p* = 0.038) and decreased subsequently, although levels after 120 min remained higher than baseline (post-hoc test, *p* = 0.031; Fig. 3). Overall, interindividual variability showed that the hormonal response was not uniform or consistent (Fig. 3).

## Discussion

The present study of the holothuroid echinoderm *Cucumaria frondosa* compared the effects of reactive versus anticipatory stressors (direct predator attacks versus cues of predation risk) on behavioural, immune and hormonal markers. It revealed that chemical cues (scent) from a nearby predator or injured conspecifics triggered all markers. Cortisol spiked within 30 min and cell counts in the hydrovascular fluid increased steadily over 3 h to levels up to 700–770% greater than baseline levels measured in undisturbed individuals. In addition, chronic exposure to contactless stress for 3 days eventually led to a decrease in the immune markers, providing evidence of something akin to habituation^40^. Such findings of adaptive stress responses also intersect with two transformative fields of research; one seeking to explore analogues of emotions in non-vertebrates through behavioural, neural and physiological approaches^8^, and the other devoted to the link between emotional and immune states in animals, mainly humans^18,41^. The anticipatory immune response to imminent threat seen here in a holothuroid, paralleling hormonal and behavioural responses, adds to recent breakthroughs on analogues of anxiety reported in other phyla (recently reviewed^8^). Because echinoderms are the closest non-chordate invertebrate clade to mammals/humans^38^, data on their cognitive-like processes offer interesting perspectives in the study of anxiety and other stress responses.

A substantial body of literature on brain circuitry and other aspects of neuroscience have historically drawn from studies of invertebrate biology and physiology^42^, while research on cognitive phenomena in invertebrates is gaining interest^8,43^. A common framework for the study of emotions across species, including invertebrates, has also been recently proposed^10^. To date, studies on invertebrate models have typically centred on species with a distinct cephalic region (including two eyes) and recognizable behavioural reactions (agitation), such as bees, flies, crabs and snails. Echinoderms have remained largely unstudied despite their pivotal position in the deuterostome clade, possibly due to technical difficulties; they have no head and their nervous system is among the least studied^44^. Work on learning abilities in echinoderms is also scarce^45,46^ and it was recently suggested that this paucity of information prevented a clear understanding of the role of neural centralization in the evolution of associative learning^47^. It was also recently demonstrated that even single-celled organisms can display simple learning^48^.

Moving away from anthropocentric analogy, the unifying framework proposed in 2014 to study emotions across species^10^ outlined behavioural, neural and physiological (including hormonal) underpinnings of central emotional states. The building blocks of emotional responses, which set them apart from simple reflexes, were defined as scalability (intensity), valence (antithesis), persistence and generalization^10^. While humoral/cellular immunity pathways were not covered in that study, the responses measured here in *C. frondosa* possess some of the features of these building blocks, such as scalability and persistence. The nature of the signal clearly influenced the intensity of the response, i.e. maximum upon direct contact with the predator, mildest when only the scent of a predator was perceptible, and intermediate when injured conspecifics could be sensed (evoking alarm signals triggered by active predatory events^49^). These results are also akin to stimulus decoupling in humans, which involves the anticipation or recollection of a stimulus instead of a direct confrontation with it, exemplified by the direct versus anticipated encounter with a predator^10^. Although it was not explicitly measured here, the response of holothuroids was almost certainly persistent (lasting hours after the stimulus was applied) since the cortisol level was still elevated after 3 h and coelomocyte counts take a long time to decrease, based on a previous study^31^. Valence is more difficult to assess since opposites states are hard to define in holothuroids beyond behavioural approach vs avoidance, for which neurobiological underpinnings have yet to be understood.

Regardless of whether the response of *C. frondosa* to direct and indirect indicators of a predator qualifies as anxiety or apprehension, it belies anticipation of injury by triggering an immune reaction. This is a useful adaptive response because, while the escape behaviour of *C. frondosa* is often successful, sublethal predation by the asteroid *S. endeca* can generate lesions on the body wall. In exposures to predator scent only, the counts of phagocytes rose first, whereas morula cells and hemocytes spiked only after 150–180 min, as phagocytes counts started to decrease. Phagocytes represent the frontline of the immune response; they are primarily designed to engulf pathogens or dead cells^31^. Morula cells are hypothesized to secrete humoral effectors and provide the foundation for tissue repair^50,51^, whereas hemocytes are presumed to provide oxygen and nutrients to regenerating tissues^52^, and they are also linked to packaging and oxidation of material captured in cellular aggregates^31^. After the first spike, the coelomocyte counts returned to normal when the stress became chronic (> 24 h) without materializing in terms of physical encounter/attack. Hence, to balance energetic costs with survival, the holothuroid was apparently able to assess when danger was high/imminent and react by developing an anticipatory immune reaction, which eventually waned after prolonged/repeated stimulation without direct finality. In addition, *C. frondosa* exhibited a clear (slightly more pronounced) increase in immune responses when exposed to the scent of injured conspecifics. Research has shown that both animals and plants produce secondary metabolites in response to signals from wounded neighbours^49^. The present study indicates that holothuroids can stand ready for injuries when sensing cues both from the predator and from conspecifics, helping to maximize survival at the population level.

While predator-induced stress is a long-standing field of study, novel perspectives on the ecology of fear are emerging^5^. Moreover, the link between stress and immunity in prey species has only recently been explored in non-vertebrates (i.e. insects), indicating that predation risk modulates defence against pathogens and, inversely, that immune challenge increases predation risk. In larval Lepidoptera, predator-induced stress compromised immunity to bacteria and had physiological outcomes, including reduced body mass^53^. On the other hand, immune-challenged crickets were slower to react to predators^54^. Here, the predation risk itself seemed to trigger the innate immune response of holothuroids. It would be interesting to investigate the metabolic and fitness costs of this response. Another question is whether individuals of *C. frondosa* already undergoing an immune challenge (e.g. presence of foreign particles in the hydrovascular system) would display a different (e.g. slower, incomplete) response to predation, similar to crickets^54^.

Upon exposure to a perceived threat emanating from the scent of a predator, cortisol levels in *C. frondosa* rose above baseline level measured pre-trial and in control individuals. The exact role of cortisol in holothuroids remains incompletely understood, but in vertebrates it has been shown to mobilize energy to meet excessive metabolic demands and trigger a broad range of responses working towards resumption of homeostasis^55^. The cortisol spike in *C. frondosa* paralleled the rise in coelomocyte counts, similar to what has been described in humans^56,57^. A hormonal cue for the release of coelomocytes in the hydrovascular system might thus be present. Since there was no loss of tissue integrity, the increase in both cortisol and coelomocytes evokes preparatory actions in the face of a potential injury, i.e. the holothuroid can detect an imminent attack and react to increase its chance of survival to nonlethal predatory events. The ability to stay alert to environmental cues and to react to predators (freeze, fight or flight) relies on the integration of sensory information and its translation into motor output via the nervous system^58^. Such reactions are usually ascribed to vertebrates but invertebrates may possess comparable counterparts, as recently highlighted by a review which showed that features of neuroautonomic regulation of the cardiac function appeared early in the evolution of decapod crustaceans^58^.

Future studies that could build on the present findings include assessments of how stress affects cognitive processes and appraisal of the environment, which is a fairly new branch of non-human research on emotions, dating back less than 20 years^59^, even though Darwin drew attention to it long ago^9^. The holothuroid *C. frondosa* could be used to study how individuals, whether undisturbed or recently exposed to stressful conditions that stimulated their immune responses, react to the presence of food, knowing that feeding requires the extension of vulnerable body parts (tentacles). A difference in the number of feeding individuals would provide evidence of “cognitive bias”, as recently shown in shaken bees^11–13^. Measurements of biogenic amines (e.g. dopamine, serotonin) could also be attempted. Ultimately, further research with echinoderms like *C. frondosa* may offer clues into how cognition and emotion interact in ecological contexts, how the simplest neural networks can underpin complex assessments and responses, and how central emotion states evolved in higher deuterostomes, all the way to humans. More pragmatic outcomes include a refined understanding of stress, pain and fear in non-vertebrate species, ultimately leading to the design of suitable animal care protocols.

## Methods

### Choice of focal species

The sea cucumber *Cucumaria frondosa* belongs to class Holothuroidea of phylum Echinodermata, and is thus a member of the lowest deuterostome clade (sitting closer to humans than nearly all other non-vertebrates^38^). Its feeding ecology, reproduction, larval development, behavioural response to stressors and most aspects of its anatomy, general biology, biochemical composition and metabolites are well known^60^. Investigations of its immune system and how it responds to the presence of foreign particles in its tissues or cavities and to injuries have recently been published^31^.

### Collection and maintenance

Adult holothuroids (12–15 cm contracted length) were collected by divers between 10–15 m depths in eastern Newfoundland, Canada (47° 12′ 45.7″ N, 52° 50′ 41.3″ W) in January 2018 and September 2019. They were carefully detached from their substrates to avoid damaging the tissues, especially the ambulacral podia. Individuals were maintained in a 200-L tank with running unfiltered seawater (60 L h^−1^) that provided them with planktonic food (their natural diet) at ambient temperatures ranging from − 1 to 5 °C. Light was supplied to a maximum intensity of 200 lx following natural photoperiod. Only healthy individuals acclimated for a minimum of eight weeks that were firmly attached and showed no sign of stress behaviour^39^ or physical damage were used. Sex was determined based on the external sexual dimorphism of the genital papilla^61^.

Four individuals of the predatory asteroid *Solaster endeca* (about 24 cm in diameter) were collected in Bay Bulls (47° 18′ 41.8″ N, 52° 48′ 33.1″ W) in July 2018 and maintained separately under the same conditions as the holothuroids. They were fasted for a month to standardize their hunger level before the experiment^62^.

### Chronic exposures to direct and indirect stressors

#### Treatments and experimental design

Twelve 20-L tanks were dedicated to each experiment consisting of 6 header tanks flowing unidirectionally into 6 other tanks to create 6 independent paired units. All units received similar flows (about 19 L h^−1^) and were randomly oriented to minimize tank effects. Fluorescein sodium salt was used to verify that water entering the experimental tank was well mixed. In each experiment, 3 of the paired units were used for one of three treatments: (1) Direct physical contact between the holothuroids and their predator, the asteroid *S. endeca* (involving sublethal predation attempts and/or injuries); (2) exposure to the predator cues through water (contactless); and (3) exposure to cues from an injured conspecific through water. The 3 other paired units were used for controls, whereby holothuroids were exposed to natural seawater flowing from bare headers. Experimental tanks that had to be reused were emptied and scrubbed to prevent any sensory bias.

At the onset of an experiment, 6 holothuroids (3 of each sex per experimental tank) of a similar size (89.1 ± 11.7 g body-wall weight) were distributed in each of the 6 downflow tanks (i.e. 18 experimental sea cucumbers and 18 control sea cucumbers per treatment). They were left to acclimate for at least a week ^31^. In experiment 1, one asteroid was introduced in each of 3 downflow tanks alongside the holothuroids; in experiment 2 one predatory asteroid was placed in each of 3 header tanks (running into the downflow tanks); and in experiment 3, one injured holothuroid was placed in each of 3 headers. The injured individual had a 3-cm long cut in the body wall; rapid healing occurred inside 10 d, as is common in this species^60^.

#### Behavioural responses

A Brinno MAC200 camera was placed above the tanks to capture the entire experimental arena with an automated infrared light allowing recording to continue at night. A picture was taken every 30 s (aligned with the slow movements of sea cucumbers), which the camera software stitched into a video clip. Behavioural scores were established at 10-min intervals, i.e. ± 5 min around times 0, 3, 6, 24 and 72 h, based on three criteria: evidence of an escape response (defined below); displacement speed; and cycles of deployment and extension of tentacles. A score of 0 corresponded to the relaxed/resting state, i.e. no escape response, no movement, and normal tentacle state; while a score of 3 meant a full-fledged escape response, which consists of detaching from the substrate, contracting and elongating the body^63^ and undergoing active buoyancy adjustment (ABA)^39^. See Table 1 for detailed definitions. Scores of individuals inside a tank were averaged, and the proportion of individuals with visible mucus was also noted.

**Table 1:** Behavioural scores used in the study of *Cucumaria frondosa*.

Score	Definition
0	Normal relaxed posture (not bloated), podia extended, firmly attached to substrate, tentacles extended or retracted, no displacement
1	Tensed posture (slightly bloated), podia extended, partly detached from substrate, tentacles extended or retracted, slow movement (crawling)
2	Tensed posture (slightly bloated), podia retracted, detached from substrate, tentacles retracted or limp, fast movement (rolling or crawling)
3	Fully developed active buoyancy adjustment reaction (as per Hamel et al. 2019); body nearly round (bloated), podia retracted, detached from substrate, tentacles retracted, severe contractions (peristaltic motion), fast movement (rolling, bouncing)

#### Sample collection and analysis of coelomocytes

One holothuroid was collected from each tank after 0, 3, 6, 24 and 72 h (terminal procedure). It was opened dorsally using a scalpel, avoiding the radial canals and row of ambulacral podia, keeping the Polian vesicle intact. The latter holds uncontaminated hydrovascular fluid in *C. frondosa*^31^. A small opening at the base of the Polian vesicle allowed the entire fluid to leak into 50-ml Falcon tubes. The fluid was immediately mixed and inverted six times to resuspend and homogenize any free coelomocytes. A subsample of 1 ml was collected and placed into a Neubauer counting chamber, which was examined under a light microscope. Images were taken at 400 × using an Olympus DP73 digital camera to count and identify coelomocytes in grid cells selected using a random integer set generator (random.org). For each time point, the dominant coelomocyte types (phagocytes, morula, hemocytes) and the coelomocyte count (all types pooled) were scored in 3–4 replicate cells. The mean density of coelomocyte (ml^−1^) established from replicates was compared among treatments over time.

### Short-term contactless exposure to predator

The short-term experiment (3 h) was conducted using the same general set up as the chronic exposures outlined earlier. It tested contactless exposure to the predator only, and it was monitored directly rather than through video analysis. One holothuroid per experimental tank was assessed after 0, 30, 60, 90, 120, 150 and 180 min, along with controls at the first and last time points. The cloacal opening (respiration) and the force of attachment to the substrate were established at each time point. These parameters are behavioural indicators of the level of stress in holothuroids^39,64^. The number of cloacal openings inside a period of 2 min was measured three times in close succession. Attachment strength was measured by wrapping a zip-tie around the middle of each individual, attaching the loop to a hand-held precision spring balance, and measuring the force required to perpendicularly pull the individual off^39^.

After the above assessment, individuals were sampled (terminally) for analysis of free coelomocytes as described for the chronic exposures. In addition, cortisol levels were measured. For the latter, 1-ml samples of Polian vesicle fluid were placed in vials and frozen undiluted at -80 °C within 10 min of collection. For processing, the fluid was thawed, and the pH lowered to 1.5–2.0 using 0.5 M HCL. Samples were washed once with 4 ml of dichloromethane following the procedure provided for a competitive cortisol ELISA assay (Cayman Chemical 500360). Dichloromethane was evaporated out of the washed sample using a nitrogen stream. Preparation of assay-specific reagents followed standard ELISA kit protocol. Before plating, each sample was centrifuged at 4000 rpm for 5 min. The incubation and development of the plate was completed as per vendor instructions. The 8-point standard curve, blank, total activity, non-specific binding and maximum binding wells were all run in duplicate. The plate was shaken mechanically for 3 s and read at 420 nm using a microplate reader (Molecular Devices SpectraMax® M5) and the software SoftMax® Pro v6.4. Data were processed using the publicly available Excel program designed for this ELISA kit.

### Data analysis

In chronic exposure trials, two-way analysis of variance (ANOVA) followed by pairwise comparison (Holm-Sidak) was used to assess the effect of treatment (contact, contactless, injured conspecific; and their three respective controls) and time (0, 3, 6, 24, 72 h) on the dependent variables (coelomocyte densities and behaviour scores). Because controls for both variables (representing baseline metrics) were not statistically different across treatments at any time point (Holm-Sidak, *p* > 0.20), they were pooled to generate four treatment categories (i.e. contact, contactless, injured conspecifics, control). To subsequently deal with interactions between treatment and time, analyses were conducted at each level of the two factors using one-way ANOVA or ANOVA on ranks, depending on data homoscedasticity, followed by pairwise comparisons with Holm-Sidak or Dunn’s methods, respectively. In the short-term contactless exposure trials, cortisol levels over time were not normally distributed and were analysed using a one-way ANOVA on ranks followed by Dunn’s pairwise tests. All tests used *α* = 0.05, although the analyses were interpreted cautiously, following calls to move away from arbitrary thresholds (including by the American Statistical Association^65^); hence, the principles of statistical clarity^66^ were followed.

## Data Availability

The datasets generated during and/or analysed during the current study are available from the corresponding author on reasonable request.
